# Subtypes and onset of hypertensive disorders of pregnancy and cardiovascular disease within 5 years after delivery

**DOI:** 10.3389/fcvm.2026.1701507

**Published:** 2026-02-19

**Authors:** Hui Hu, David A. Savitz, Elizabeth A. Shenkman

**Affiliations:** 1 Channing Division of Network Medicine, Brigham and Women’s Hospital and Harvard Medical School, Boston, MA, United States; 2 Department of Epidemiology, Brown University School of Public Health, Providence, RI, United States; 3 Department of Health Outcomes and Biomedical Informatics, College of Medicine, University of Florida, Gainesville, FL, United States

**Keywords:** arrhythmia, cardiac arrest, cardiomyopathy, cardiovascular diseases, cerebrovascular disease, eclampsia, gestational hypertension, heart failure

## Abstract

**Background:**

Hypertensive disorders of pregnancy (HDP) are established predictors of long-term cardiovascular disease (CVD), but the short-term postpartum CVD risk by HDP subtype and onset remains unclear.

**Materials and methods:**

We linked electronic health records with vital statistics for 755,606 singleton deliveries in Florida (2012–2017) to examine how different subtypes and onset of HDP are associated with CVD within 5 years postpartum. We classified HDP into six subtypes—chronic hypertension, gestational hypertension, mild preeclampsia, severe preeclampsia, eclampsia, and superimposed preeclampsia - and defined onset as early (<34 weeks) or late (≥34 weeks). Seven CVD outcomes (heart failure, ischemic heart disease, cerebrovascular disease/stroke, arrhythmia/cardiac arrest, cardiomyopathy, peripheral vascular disease, and new-onset chronic hypertension) within five years postpartum were identified. Cox proportional hazards models estimated hazard ratios (HRs) and 95% confidence intervals (CIs) after adjusting for sociodemographic and clinical covariates.

**Results:**

Compared with normotensive pregnancies, superimposed preeclampsia carried the highest risks for stroke (HR 3.39; 95% CI 2.93–3.92), arrhythmia (2.62; 2.25–3.07), and peripheral vascular disease (3.09; 2.67–3.57). Eclampsia showed the strongest associations with heart failure (5.23; 3.70–7.39), ischemic heart disease (3.61; 2.65–4.92), and cardiomyopathy (5.25; 3.37–8.18). Severe preeclampsia was most strongly associated with new hypertension (3.27; 3.10–3.46). Early-onset eclampsia and superimposed preeclampsia showed higher CVD risks than their late-onset counterparts, whereas late-onset gestational hypertension and mild preeclampsia were more strongly associated with new hypertension and cardiomyopathy, respectively.

**Conclusions:**

HDP subtypes and onset timing impart distinct CVD risk profiles within five years postpartum.

## Introduction

Cardiovascular disease (CVD) is the leading cause of death in women worldwide (1). Pregnancy is now understood as a natural “stress test” for the cardiovascular system, and adverse pregnancy outcomes can unmask latent cardiometabolic risk factors (2). Hypertensive disorders of pregnancy (HDP)—which complicate approximately 13%–15% of all pregnancies in the US (3)—have emerged as significant sex-specific risk factors for future maternal CVD (4–8). Despite the well-established link between HDP and long-term CVD (5, 7–11), relatively little is known about how soon after pregnancy these cardiovascular risks become apparent (12). Most research to date has focused on outcomes decades after an affected pregnancy, and data on the early postpartum period remains limited (13). Emerging evidence suggests that cardiovascular sequelae may manifest earlier than previously thought (12). However, much of the existing literature on short-term postpartum risk is restricted to the first 6–12 months after delivery, leaving a critical knowledge gap regarding cardiovascular outcomes in the subsequent few years following HDP (14). Findings from the few studies focusing on short-term CVD outcomes over 1 year postpartum showed that HDP are associated with a 1.4- to 2.9-fold increased risk of CVD within 24 months postpartum (15), 1.2- to 1.4-fold increased risk of CVD within the first 5 years postpartum (16), and 2.2- to 3.3-fold increased risk of CVD hospitalizations over a mean follow-up of 7.8 years (17). These findings underscore the initial postpartum years—particularly the first 5 years after an HDP-affected pregnancy—as a critical window for detecting incipient CVD in groups of women at markedly elevated risk and implementing prevention strategies (14).

Another important knowledge gap is the limited granularity of prior studies regarding which HDP subtypes and onset confer the greatest cardiovascular risk in the postpartum period. Previous studies mainly focused on preeclampsia, and found that women with a history of preeclampsia have around 2 times higher risk of CVD compared with those without preeclampsia (7–9). Meta-analyses showed that the strengths of the associations vary by specific CVD outcomes, ranging from 1.8- to 6.7-fold increased risk for stroke and future hypertension, respectively (5, 7, 9–11). A dose-response relationship has also been observed between the severity of preeclampsia and long-term maternal CVD risk (8, 18, 19), with relative risks increased from 2.0 to 5.4 observed for mild and severe preeclampsia, respectively (8). The associations between gestational hypertension and maternal CVD risk were less studied, and it is generally believed that compared with preeclampsia, the association between gestational hypertension and future CVD risk is weaker (5, 18, 19). However, several recent studies found similar or higher CVD risk associated with gestational hypertension compared with preeclampsia (11, 20, 21). These observations underscore that HDP is not a singular entity but a spectrum of conditions, each of which may impart a distinct CVD risk profile. However, as previous studies often focus exclusively on preeclampsia or grouping all HDP together, without considering HDP subtypes and onset, it remains unclear whether relatively milder conditions like gestational hypertension carry significantly different short-term CVD risks compared with more severe subtypes like severe preeclampsia, eclampsia, or chronic hypertension with superimposed preeclampsia. In addition, most existing studies relied on administrative datasets such as insurance claims and birth registries to identify HDP and outcomes, which can introduce misclassifications and often lack clinical details such as blood pressure measures, laboratory findings, and medication prescriptions needed to distinguish HDP subtypes and onset (22). These limitations hinder the ability to tailor postpartum surveillance and counseling according to HDP subtypes and onset.

To address these gaps, in the present study, we leveraged a robust, statewide linked data resource from Florida that combines electronic health records (EHR) with vital statistics birth and fetal death records to examine associations between HDP subtypes and onset of CVD outcomes within 5 years after delivery.

## Methods

### Study population

We conducted a retrospective cohort study leveraging a unique statewide linked EHR-vital statistics birth and fetal death records dataset, with individual-level linked data from the 2012–2018 Florida Vital Statistics Birth Records (VSBR) and Vital Statistics Fetal Death Records (VSFDR), obtained from the Bureau of Vital Statistics, Florida Department of Health (http://www.floridahealth.gov/certificates/), and EHR data (2012–2024) from the OneFlorida + Clinical Research Network (23). The data linkage was performed using a privacy-preserving record linkage tool provided by Datavant based on personal identifiers including individuals' name, date of birth, sex, and 5-digit ZIP Code. To account for changes of women's last name, we separately performed linkages using both women's maiden name and current last name at delivery as recorded in the VSBR and VSFDR data.

Conception date was calculated by delivery date and gestation age, and last menstrual period was used to estimate conception date when gestation age was missing. Gestational timing variables were used to establish a consistent pregnancy timeline for linkage across data sources and to define HDP onset. A total of 779,591 pregnancies with a conception date between January 1st, 2012 and December 31st, 2017 were included in the linked EHR-VSBR/VSFDR data. We excluded women with multiple pregnancies (*n* = 23,654) due to their increased risk of HDP and CVD compared with those with singleton pregnancies (24). We also excluded those with erroneously coded gestational ages (i.e., <20 or >45 weeks, *n* = 331), yielding 755,606 pregnancies.

### Assessment of HDP subtypes and onset

Information from both the EHR (i.e., diagnoses, blood pressure measures, medication prescriptions, and laboratory test results) and VSBR/VSFDR were used to determine HDP subtypes, including chronic hypertension, gestational hypertension, mild preeclampsia, severe preeclampsia, eclampsia, superimposed preeclampsia on chronic hypertension, and unspecified HDP. Supplementary Table S1 shows the International Classification of Diseases, Ninth/Tenth Revision, Clinical Modification (ICD-9-CM/ICD-10-CM) codes used to identify HDP subtypes from the EHR data. Proteinuria was determined based on results of relevant laboratory tests performed between the 20th week of gestation and delivery. Supplementary Table S2 shows the LOINC Codes and cut points used to determine proteinuria. Prescriptions of antihypertensive medications were identified using relevant RxNorm Concept Unique Identifiers (RxCUIs) compiled in a previous study (25). Supplementary Table S3 shows the rule-based phenotyping algorithm to determine HDP subtypes, modified based on a recently developed algorithm to leverage information from both the EHR and VSBR/VSFDR data (26). Onset of HDP (except for chronic hypertension) was categorized as early onset (<34th week of gestation) and late onset (≥34th week of gestation) (27).

### Assessment of CVD outcomes

CVD outcomes were determined based on ICD-9-CM/ICD-10-CM codes using the EHR data (Supplementary Table S1). CVD onset was assessed by the first presence of relevant diagnoses. The CVD outcomes include heart failure, ischemic heart disease, cerebrovascular disease or stroke, arrhythmia or cardiac arrest, cardiomyopathy, peripheral vascular disease with onset within 5 years after delivery, as well as new chronic hypertension with onset from 43 days after delivery to 5 years after delivery (since hypertension within 42 days after delivery is considered an HDP) (28).

### Covariates

A number of covariates were considered and determined based on both the EHR and VSBR/VSFDR data, including age at delivery (<20, 20–24, 25–29, 30–34, 35–39, and ≥40 years old), race/ethnicity (non-Hispanic White, non-Hispanic Black, Hispanic, Asian/Pacific Islander, and others), education (<high school, high school or equivalent, and >high school), marital status at delivery (yes or no), pre-pregnancy BMI (underweight: <18.5 kg/m^3^, normal: 18.5–24.9 kg/m^3^, overweight: 25–29.9 kg/m^3^, obese: ≥30 kg/m^3^), smoking during pregnancy (yes or no), prenatal care began (no care, started in the first trimester, started in the second trimester, started in the third trimester, and yes but unknown start date), insurance type (Medicaid, private insurance, self-pay, and others), US Department of Agriculture's nutrition program for Women, Infants, and Children (WIC: received or not), gestational diabetes mellitus (GDM: yes or no), pre-pregnancy depression (yes or no), prenatal depression (yes or no), parity (nulliparous or non-nulliparous), and year of conception.

### Statistical analyses

Maternal characteristics and HDP onset as well as CVD outcomes and onset by HDP subtype were described. Cox proportional hazards models were used to examine the associations between HDP subtypes and onset and CVD within 5 years after delivery. Hazard ratios (HRs) and 95% confidence intervals (CIs) for the time to first diagnosis for each of the seven outcomes within 5 years after delivery were reported. For all the seven CVD outcomes, we excluded women with existing CVD outcomes (except for chronic hypertension) on or before the date of delivery (*n* = 13,411), and for new chronic hypertension, we further excluded those with hypertension onset on or before 42 days after delivery (*n* = 74,333). Observations were censored upon loss of EHR coverage (i.e., based on the enrollment information in EHR), at the start of the next pregnancy, or 5 years after delivery, whichever came first. We used inverse probability of treatment-weighted Cox proportional hazard models to visualize cumulative hazard curves (29). All time-to-event analyses used days since delivery as the timescale.

All statistical analyses were conducted using R 4.4.2 [RStudio Team (2020)]. This study was approved by institutional review boards at the Mass General Brigham (2021P002672), Florida Department of Health (2019–106), and the University of Florida (IRB201902383).

## Results

Maternal characteristics by HDP subtype are presented in Table 1. Among the 755,606 singleton pregnancies with a conception date between 2012 and 2017, a total of 146,534 HDP cases were identified, including chronic hypertension (*n* = 59,828, 7.9%), gestational hypertension (*n* = 38,806, 5.1%), mild preeclampsia (*n* = 11,480, 1.5%), severe preeclampsia (*n* = 10,857, 1.4%), eclampsia (*n* = 1,736, 0.2%), superimposed preeclampsia on chronic hypertension (*n* = 19,045, 2.5%), and unspecified HDP (*n* = 4,782, 0.6%). Compared with pregnancies without HDP, those affected by HDP were more likely to occur in women who were older, non-Hispanic Black, obese before pregnancy, and publicly insured. The prevalence of HDP increased notably with maternal age, particularly for chronic hypertension and superimposed preeclampsia on chronic hypertension, which were most common among women aged ≥35 years. Racial and ethnic disparities were evident, with non-Hispanic Black women representing a disproportionate share of cases with chronic hypertension, severe preeclampsia, eclampsia, and superimposed preeclampsia. Women with HDP were also more likely to have lower educational attainment, be unmarried, initiate prenatal care later, and have GDM, pre-pregnancy depression, and prenatal depression. Among HDP subtypes, those with chronic hypertension and superimposed preeclampsia had the highest rates of obesity, GDM, and prenatal depression. Notably, women with eclampsia were more likely to be younger (<25 years), non-Hispanic Black, and enrolled in WIC.

**Table 1:** Maternal characteristics, HDP onset, and CVD outcomes and onset by HDP subtypes among singleton pregnancies with a conception date 2012–2017 in the EHR-vital statistics birth and fetal death records data in Florida (*n* = 755,606).

Characteristics	Total	No HDP	Chronic hypertension	Gestational hypertension	Mild preeclampsia	Severe preeclampsia	Eclampsia	Superimposed preeclampsia on chronic hypertension	Unspecified HDP
	(*n* = 755,606)	(*n* = 609,072)	(*n* = 59,828)	(*n* = 38,808)	(*n* = 11,479)	(*n* = 10,856)	(*n* = 1,736)	(*n* = 19,045)	(*n* = 4,782)
Maternal age at delivery									
<20	60,081 (8.0)	49,638 (8.1)	2,594 (4.3)	3,575 (9.2)	1,373 (12.0)	1,303 (12.0)	254 (14.6)	976 (5.1)	368 (7.7)
20–24	212,602 (28.1)	175,219 (28.8)	13,705 (22.9)	10,777 (27.8)	3,764 (32.8)	3,183 (29.3)	517 (29.8)	3,975 (20.9)	1,462 (30.6)
25–29	223,523 (29.6)	181,741 (29.8)	17,578 (29.4)	11,173 (28.8)	3,176 (27.7)	2,727 (25.1)	447 (25.7)	5,254 (27.6)	1,427 (29.8)
30–34	162,287 (21.5)	129,315 (21.2)	14,721 (24.6)	8,200 (21.1)	1,993 (17.4)	2,095 (19.3)	315 (18.1)	4,696 (24.7)	952 (19.9)
35–39	77,838 (10.3)	59,432 (9.8)	8,641 (14.4)	4,018 (10.4)	916 (8.0)	1,194 (11.0)	158 (9.1)	3,037 (15.9)	442 (9.2)
≥40	19,275 (2.6)	13,727 (2.3)	2,589 (4.3)	1,065 (2.7)	257 (2.2)	354 (3.3)	45 (2.6)	1,107 (5.8)	131 (2.7)
Race/ethnicity									
Non-Hispanic White	283,178 (37.5)	229,311 (37.6)	20,968 (35.0)	17,019 (43.9)	4,433 (38.6)	3,366 (31.0)	545 (31.4)	5,641 (29.6)	1,895 (39.6)
Non-Hispanic Black	177,404 (23.5)	131,141 (21.5)	21,274 (35.6)	9,497 (24.5)	2,947 (25.7)	3,419 (31.5)	614 (35.4)	7,236 (38.0)	1,276 (26.7)
Hispanic	258,679 (34.2)	217,537 (35.7)	15,606 (26.1)	10,619 (27.4)	3,702 (32.3)	3,633 (33.5)	520 (30.0)	5,605 (29.4)	1,457 (30.5)
Asian/Pacific Islander	15,412 (2.0)	13,624 (2.2)	576 (1.0)	676 (1.7)	128 (1.1)	163 (1.5)	14 (0.8)	188 (1.0)	43 (0.9)
Other	15,916 (2.1)	13,257 (2.2)	1,048 (1.8)	825 (2.1)	202 (1.8)	211 (1.9)	29 (1.7)	260 (1.4)	84 (1.8)
Missing	5,017 (0.7)	4,202 (0.7)	356 (0.6)	172 (0.4)	67 (0.6)	64 (0.6)	14 (0.8)	115 (0.6)	27 (0.6)
Education									
<High school	118,774 (15.7)	95,908 (15.7)	9,625 (16.1)	5,432 (14.0)	1,929 (16.8)	1,869 (17.2)	355 (20.4)	2,874 (15.1)	782 (16.4)
High school or equivalent	294,461 (39.0)	235,196 (38.6)	24,525 (41.0)	14,605 (37.6)	4,846 (42.2)	4,308 (39.7)	726 (41.8)	8,253 (43.3)	2,002 (41.9)
> High school	335,958 (44.5)	272,847 (44.8)	25,110 (42.0)	18,504 (47.7)	4,615 (40.2)	4,581 (42.2)	632 (36.4)	7,719 (40.5)	1,950 (40.8)
Missing	6,413 (0.8)	5,121 (0.8)	568 (0.9)	267 (0.7)	89 (0.8)	98 (0.9)	23 (1.3)	199 (1.0)	48 (1.0)
Marital Status									
Not married	469,116 (62.1)	375,065 (61.6)	38,264 (64.0)	23,827 (61.4)	7,932 (69.1)	7,103 (65.4)	1,271 (73.2)	12,490 (65.6)	3,164 (66.2)
Married	285,415 (37.8)	233,254 (38.3)	21,391 (35.8)	14,948 (38.5)	3,542 (30.9)	3,720 (34.3)	457 (26.3)	6,499 (34.1)	1,604 (33.5)
Missing	1,075 (0.1)	753 (0.1)	173 (0.3)	33 (0.1)	5 (0.0)	33 (0.3)	8 (0.5)	56 (0.3)	14 (0.3)
Pre-pregnancy BMI									
Underweight (<18.5)	32,491 (4.3)	28,865 (4.7)	1,310 (2.2)	1,108 (2.9)	338 (2.9)	403 (3.7)	62 (3.6)	267 (1.4)	138 (2.9)
Normal (18.5–24.9)	301,487 (39.9)	262,644 (43.1)	14,218 (23.8)	11,992 (30.9)	3,564 (31.0)	3,622 (33.4)	538 (31.0)	3,610 (19.0)	1,299 (27.2)
Overweight (25.0–29.9)	186,803 (24.7)	152,285 (25.0)	13,477 (22.5)	9,700 (25.0)	2,911 (25.4)	2,549 (23.5)	381 (21.9)	4,258 (22.4)	1,242 (26.0)
Obese (≥30.0)	188,073 (24.9)	128,840 (21.2)	26,874 (44.9)	13,175 (33.9)	4,027 (35.1)	3,393 (31.3)	588 (33.9)	9,468 (49.7)	1,708 (35.7)
Missing	46,752 (6.2)	36,438 (6.0)	3,949 (6.6)	2,833 (7.3)	639 (5.6)	889 (8.2)	167 (9.6)	1,442 (7.6)	395 (8.3)
Smoking during pregnancy									
No	696,202 (92.1)	561,867 (92.2)	53,990 (90.2)	35,904 (92.5)	10,625 (92.6)	10,278 (94.7)	1,587 (91.4)	17,636 (92.6)	4,315 (90.2)
Yes	54,820 (7.3)	43,631 (7.2)	5,434 (9.1)	2,637 (6.8)	805 (7.0)	493 (4.5)	136 (7.8)	1,251 (6.6)	433 (9.1)
Missing	4,584 (0.6)	3,574 (0.6)	404 (0.7)	267 (0.7)	49 (0.4)	85 (0.8)	13 (0.7)	158 (0.8)	34 (0.7)
Prenatal care began									
No care	13,326 (1.8)	10,538 (1.7)	1,241 (2.1)	580 (1.5)	127 (1.1)	290 (2.7)	65 (3.7)	398 (2.1)	87 (1.8)
First trimester	390,748 (51.7)	315,997 (51.9)	31,281 (52.3)	19,683 (50.7)	5,832 (50.8)	4,980 (45.9)	850 (49.0)	9,525 (50.0)	2,600 (54.4)
Second trimester	153,503 (20.3)	124,265 (20.4)	12,250 (20.5)	7,634 (19.7)	2,329 (20.3)	2,065 (19.0)	388 (22.4)	3,544 (18.6)	1,028 (21.5)
Third trimester	40,023 (5.3)	32,805 (5.4)	3,090 (5.2)	2,051 (5.3)	515 (4.5)	431 (4.0)	75 (4.3)	814 (4.3)	242 (5.1)
Yes, no clear information	155,247 (20.5)	123,297 (20.2)	11,701 (19.6)	8,743 (22.5)	2,655 (23.1)	3,034 (27.9)	342 (19.7)	4,674 (24.5)	801 (16.8)
Missing	2,759 (0.4)	2,170 (0.4)	265 (0.4)	117 (0.3)	21 (0.2)	56 (0.5)	16 (0.9)	90 (0.5)	24 (0.5)
Insurance type									
Medicaid	541,557 (71.7)	430,437 (70.7)	46,515 (77.7)	26,827 (69.1)	9,303 (81.0)	7,632 (70.3)	1,458 (84.0)	15,619 (82.0)	3,766 (78.8)
Private insurance	164,780 (21.8)	137,105 (22.5)	10,428 (17.4)	10,023 (25.8)	1,619 (14.1)	2,243 (20.7)	165 (9.5)	2,418 (12.7)	779 (16.3)
Self-pay	30,262 (4.0)	26,350 (4.3)	1,107 (1.9)	1,215 (3.1)	351 (3.1)	619 (5.7)	55 (3.2)	443 (2.3)	122 (2.6)
Others	12,037 (1.6)	9,850 (1.6)	969 (1.6)	497 (1.3)	124 (1.1)	212 (2.0)	23 (1.3)	303 (1.6)	59 (1.2)
Missing	6,970 (0.9)	5,330 (0.9)	809 (1.4)	246 (0.6)	82 (0.7)	150 (1.4)	35 (2.0)	262 (1.4)	56 (1.2)
WIC									
No	250,637 (33.2)	206,458 (33.9)	17,345 (29.0)	13,725 (35.4)	2,952 (25.7)	3,461 (31.9)	472 (27.2)	4,937 (25.9)	1,287 (26.9)
Yes	499,408 (66.1)	398,194 (65.4)	41,994 (70.2)	24,828 (64.0)	8,455 (73.7)	7,297 (67.2)	1,239 (71.4)	13,950 (73.2)	3,451 (72.2)
Missing	5,561 (0.7)	4,420 (0.7)	489 (0.8)	255 (0.7)	72 (0.6)	98 (0.9)	25 (1.4)	158 (0.8)	44 (0.9)
Gestational diabetes mellitus	70,376 (9.3)	47,283 (7.8)	9,569 (16.0)	4,933 (12.7)	1,773 (15.4)	1,436 (13.2)	262 (15.1)	4,481 (23.5)	639 (13.4)
Pre-pregnancy depression	21,518 (2.8)	14,128 (2.3)	4,574 (7.6)	1,053 (2.7)	277 (2.4)	231 (2.1)	113 (6.5)	981 (5.2)	161 (3.4)
Prenatal depression	29,797 (3.9)	20,030 (3.3)	4,999 (8.4)	1,977 (5.1)	472 (4.1)	497 (4.6)	147 (8.5)	1,400 (7.4)	275 (5.8)
Parity									
Nulliparous	290,771 (38.5)	234,210 (38.5)	14,722 (24.6)	19,900 (51.3)	6,134 (53.4)	6,471 (59.6)	822 (47.4)	6,603 (34.7)	1,909 (39.9)
Non-nulliparous	462,817 (61.3)	373,282 (61.3)	44,957 (75.1)	18,769 (48.4)	5,321 (46.4)	4,335 (39.9)	909 (52.4)	12,386 (65.0)	2,858 (59.8)
Missing	2,018 (0.3)	1,580 (0.3)	149 (0.2)	139 (0.4)	24 (0.2)	50 (0.5)	5 (0.3)	56 (0.3)	15 (0.3)
Year of conception									
2012	105,732 (14.0)	87,670 (14.4)	5,885 (9.8)	4,903 (12.6)	2,312 (20.1)	1,390 (12.8)	254 (14.6)	2,671 (14.0)	647 (13.5)
2013	127,402 (16.9)	104,213 (17.1)	8,679 (14.5)	5,714 (14.7)	2,615 (22.8)	1,680 (15.5)	241 (13.9)	3,481 (18.3)	779 (16.3)
2014	129,180 (17.1)	104,952 (17.2)	9,641 (16.1)	6,072 (15.6)	2,398 (20.9)	1,667 (15.4)	254 (14.6)	3,394 (17.8)	802 (16.8)
2015	129,146 (17.1)	103,836 (17.0)	11,108 (18.6)	6,947 (17.9)	1,380 (12.0)	1,783 (16.4)	352 (20.3)	3,001 (15.8)	739 (15.5)
2016	133,126 (17.6)	105,653 (17.3)	12,125 (20.3)	7,695 (19.8)	1,304 (11.4)	2,027 (18.7)	296 (17.1)	3,191 (16.8)	835 (17.5)
2017	131,020 (17.3)	102,748 (16.9)	12,390 (20.7)	7,477 (19.3)	1,470 (12.8)	2,309 (21.3)	339 (19.5)	3,307 (17.4)	980 (20.5)
HDP onset									
Early onset	31,406 (4.2)	0 (0.0)	0 (0.0)	14,106 (36.3)	2,138 (18.6)	2,815 (25.9)	844 (48.6)	9,966 (52.3)	1,537 (32.1)
Late onset	55,300 (7.3)	0 (0.0)	0 (0.0)	24,702 (63.7)	9,341 (81.4)	8,041 (74.1)	892 (51.4)	9,079 (47.7)	3,245 (67.9)
Not applicable	668,900 (88.5)	609,072 (100.0)	59,828 (100.0)	0 (0.0)	0 (0.0)	0 (0.0)	0 (0.0)	0 (0.0)	0 (0.0)
CVD									
Heart failure									
No	747,993 (99.0)	605,204 (99.4)	57,970 (96.9)	38,410 (99.0)	11,349 (98.9)	10,625 (97.9)	1,640 (94.5)	18,072 (94.9)	4,723 (98.8)
Onset before conception	2,030 (0.3)	987 (0.2)	738 (1.2)	61 (0.2)	11 (0.1)	24 (0.2)	15 (0.9)	186 (1.0)	8 (0.2)
Onset during pregnancy	1,820 (0.2)	1,010 (0.2)	300 (0.5)	79 (0.2)	35 (0.3)	84 (0.8)	35 (2.0)	265 (1.4)	12 (0.3)
Onset after delivery	3,763 (0.5)	1,871 (0.3)	820 (1.4)	258 (0.7)	84 (0.7)	123 (1.1)	46 (2.6)	522 (2.7)	39 (0.8)
Ischemic heart disease									
No	747,531 (98.9)	604,808 (99.3)	57,774 (96.6)	38,396 (98.9)	11,352 (98.9)	10,709 (98.6)	1,663 (95.8)	18,128 (95.2)	4,701 (98.3)
Onset before conception	1,011 (0.1)	411 (0.1)	401 (0.7)	44 (0.1)	6 (0.1)	7 (0.1)	10 (0.6)	123 (0.6)	9 (0.2)
Onset during pregnancy	907 (0.1)	450 (0.1)	250 (0.4)	49 (0.1)	12 (0.1)	22 (0.2)	14 (0.8)	105 (0.6)	5 (0.1)
Onset after delivery	6,157 (0.8)	3,403 (0.6)	1,403 (2.3)	319 (0.8)	109 (0.9)	118 (1.1)	49 (2.8)	689 (3.6)	67 (1.4)
Cerebrovascular disease or stroke									
No	748,009 (99.0)	604,686 (99.3)	58,321 (97.5)	38,238 (98.5)	11,360 (99.0)	10,630 (97.9)	1,647 (94.9)	18,412 (96.7)	4,715 (98.6)
Onset before conception	2,030 (0.3)	416 (0.1)	282 (0.5)	38 (0.1)	10 (0.1)	6 (0.1)	5 (0.3)	69 (0.4)	5 (0.1)
Onset during pregnancy	1,820 (0.1)	2,343 (0.4)	765 (1.3)	386 (1.0)	65 (0.6)	154 (1.4)	65 (3.7)	313 (1.6)	41 (0.9)
Onset after delivery	3,763 (0.5)	1,627 (0.3)	460 (0.8)	146 (0.4)	44 (0.4)	66 (0.6)	19 (1.1)	251 (1.3)	21 (0.4)
Arrhythmia/Cardiac arrest									
No	750,915 (99.4)	606,410 (99.6)	58,701 (98.1)	38,525 (99.3)	11,409 (99.4)	10,761 (99.1)	1,697 (97.8)	18,674 (98.1)	4,738 (99.1)
Onset before conception	929 (0.1)	414 (0.1)	379 (0.6)	37 (0.1)	5 (0.0)	9 (0.1)	4 (0.2)	73 (0.4)	8 (0.2)
Onset during pregnancy	959 (0.1)	535 (0.1)	206 (0.3)	91 (0.2)	14 (0.1)	26 (0.2)	14 (0.8)	61 (0.3)	12 (0.3)
Onset after delivery	2,803 (0.4)	1,713 (0.3)	542 (0.9)	155 (0.4)	51 (0.4)	60 (0.6)	21 (1.2)	237 (1.2)	24 (0.5)
Cardiomyopathy									
No	751,749 (99.5)	607,311 (99.7)	58,778 (98.2)	38,575 (99.4)	11,412 (99.4)	10,725 (98.8)	1,690 (97.4)	18,502 (97.1)	4,756 (99.5)
Onset before conception	823 (0.1)	284 (0.0)	401 (0.7)	29 (0.1)	5 (0.0)	9 (0.1)	5 (0.3)	86 (0.5)	4 (0.1)
Onset during pregnancy	746 (0.1)	320 (0.1)	176 (0.3)	62 (0.2)	8 (0.1)	27 (0.2)	17 (1.0)	131 (0.7)	5 (0.1)
Onset after delivery	2,288 (0.3)	1,157 (0.2)	473 (0.8)	142 (0.4)	54 (0.5)	95 (0.9)	24 (1.4)	326 (1.7)	17 (0.4)
Peripheral vascular disease									
No	751,442 (99.4)	606,802 (99.6)	58,745 (98.2)	38,603 (99.5)	11,417 (99.5)	10,796 (99.4)	1,704 (98.2)	18,633 (97.8)	4,742 (99.2)
Onset before conception	1,053 (0.1)	487 (0.1)	386 (0.6)	51 (0.1)	7 (0.1)	5 (0.0)	10 (0.6)	100 (0.5)	7 (0.1)
Onset during pregnancy	469 (0.1)	254 (0.0)	121 (0.2)	21 (0.1)	6 (0.1)	9 (0.1)	6 (0.3)	45 (0.2)	7 (0.1)
Onset after delivery	2,638 (0.3)	1,529 (0.3)	576 (1.0)	133 (0.3)	49 (0.4)	46 (0.4)	16 (0.9)	267 (1.4)	26 (0.5)
Hypertension (>42 days after delivery)	32,205 (4.8)	24,960 (4.1)	-	3,732 (9.6)	1,345 (11.7)	1,467 (13.5)	173 (10.0)	-	577 (12.1)

Table 1 also shows HDP onset and CVD outcomes by HDP subtype. Among the 86,706 pregnancies with HDP (except for chronic hypertension), 31,406 (36.2%) had early-onset HDP, with the highest proportion observed in cases of superimposed preeclampsia (52.3%), followed by eclampsia (48.6%) and gestational hypertension (36.3%). CVD outcomes and onset varied substantially by HDP subtype. Overall, pregnancies with HDP were more likely to have each of the 7 CVD outcomes compared with those without HDP. Notably, pregnancies with superimposed preeclampsia had the highest burden of heart failure (2.7%), ischemic heart disease (3.6%), cerebrovascular disease or stroke (1.3%), arrhythmia or cardiac arrest (1.2%), cardiomyopathy (1.7%), and peripheral vascular disease (1.4%) within 5 years after delivery, followed by those with eclampsia and chronic hypertension. In addition, pregnancies with severe preeclampsia (13.5%) were most likely to have new-onset chronic hypertension after delivery, followed by those with unspecified HDP (12.1%), mild preeclampsia (11.7%), and eclampsia (10.0%).

Supplementary Figure S1 shows the cumulative hazard curves for CVD outcomes within 5 years after delivery by HDP subtype, and Table 2 presents the hazard ratios (HRs) and 95% confidence intervals (CIs). Compared with pregnancies without HDP, those with HDP had significantly elevated risks for all seven CVD outcomes. Notably, women with superimposed preeclampsia on chronic hypertension exhibited the highest HRs for cerebrovascular disease or stroke [adjusted HR (aHR): 3.39, 95% CI: 2.93, 3.92], arrhythmia or cardiac arrest (aHR: 2.62, 95% CI: 2.25, 3.07), and peripheral vascular disease (aHR: 3.09, 95% CI: 2.67, 3.57). Eclampsia was most strongly associated with heart failure (aHR: 5.23, 95% CI: 3.70, 7.39), ischemic heart disease (aHR: 3.61, 95% CI: 2.65, 4.92) and cardiomyopathy (aHR: 5.25, 95% CI: 3.37, 8.18), while severe preeclampsia was associated with the greatest risk of new-onset chronic hypertension (aHR: 3.27, 95% CI: 3.10, 3.46).

**Table 2:** Hazard ratios (HRs) and 95% confidence intervals (95% CIs) of CVD outcomes within 5 years after delivery by HDP subtype among singleton pregnancies with a conception date between 2012 and 2017 in the linked EHR-vital statistics birth and fetal death records data in Florida.

Outcome	HR (95% CI)							
	No HDP	Chronic hypertension	Gestational hypertension	Mild preeclampsia	Severe preeclampsia	Eclampsia	Superimposed preeclampsia on chronic hypertension	Unspecified HDP
Heart failure								
Crude	Ref	3.95 (3.62, 4.31)	2.07 (1.80, 2.37)	2.32 (1.86, 2.90)	3.75 (3.11, 4.51)	6.66 (4.72, 9.40)	7.89 (7.11, 8.75)	2.37 (1.69, 3.33)
Adjusted	Ref	2.68 (2.45, 2.94)	1.86 (1.62, 2.13)	2.04 (1.63, 2.56)	3.35 (2.78, 4.04)	5.23 (3.70, 7.39)	5.05 (4.53, 5.63)	1.96 (1.39, 2.75)
Ischemic heart disease								
Crude	Ref	3.50 (3.27, 3.74)	1.40 (1.24, 1.58)	1.61 (1.32, 1.96)	1.85 (1.53, 2.24)	4.40 (3.24, 5.99)	5.45 (4.99, 5.95)	2.35 (1.83, 3.02)
Adjusted	Ref	2.27 (2.11, 2.43)	1.27 (1.13, 1.44)	1.52 (1.25, 1.85)	1.78 (1.47, 2.16)	3.61 (2.65, 4.92)	3.50 (3.20, 3.84)	1.95 (1.52, 2.51)
Cerebrovascular disease or stroke								
Crude	Ref	2.50 (2.24, 2.78)	1.35 (1.13, 1.61)	1.39 (1.02, 1.88)	2.21 (1.72, 2.84)	3.50 (2.14, 5.72)	4.31 (3.75, 4.95)	1.55 (1.00, 2.41)
Adjusted	Ref	1.89 (1.69, 2.12)	1.26 (1.05, 1.50)	1.45 (1.07, 1.96)	2.00 (1.55, 2.57)	2.84 (1.73, 4.65)	3.39 (2.93, 3.92)	1.38 (0.89, 2.15)
Arrhythmia or cardiac arrest								
Crude	Ref	2.59 (2.33, 2.88)	1.35 (1.13, 1.60)	1.47 (1.10, 1.96)	1.87 (1.43, 2.44)	2.54 (1.44, 4.48)	3.46 (2.97, 4.02)	1.80 (1.20, 2.69)
Adjusted	Ref	1.94 (1.73, 2.16)	1.29 (1.09, 1.54)	1.39 (1.04, 1.87)	1.82 (1.39, 2.38)	2.06 (1.17, 3.64)	2.62 (2.25, 3.07)	1.58 (1.06, 2.37)
Cardiomyopathy								
Crude	Ref	3.72 (3.32, 4.17)	1.90 (1.59, 2.27)	2.49 (1.89, 3.28)	4.38 (3.51, 5.46)	6.54 (4.20, 10.17)	7.48 (6.54, 8.56)	1.58 (0.93, 2.68)
Adjusted	Ref	2.53 (2.25, 2.85)	1.71 (1.43, 2.06)	2.30 (1.74, 3.03)	3.90 (3.12, 4.87)	5.25 (3.37, 8.18)	4.83 (4.19, 5.57)	1.33 (0.78, 2.25)
Peripheral vascular disease								
Crude	Ref	3.22 (2.91, 3.56)	1.35 (1.13, 1.62)	1.55 (1.15, 2.09)	1.58 (1.16, 2.14)	3.05 (1.77, 5.26)	4.62 (4.02, 5.31)	2.16 (1.46, 3.18)
Adjusted	Ref	2.29 (2.06, 2.55)	1.30 (1.08, 1.56)	1.36 (1.01, 1.83)	1.60 (1.17, 2.17)	2.60 (1.50, 4.49)	3.09 (2.67, 3.57)	1.84 (1.25, 2.72)
Hypertension								
Crude	Ref	-	2.43 (2.34, 2.51)	2.94 (2.78, 3.10)	3.52 (3.34, 3.72)	2.46 (2.11, 2.87)	-	3.01 (2.77, 3.27)
Adjusted	Ref	-	2.21 (2.13, 2.29)	2.56 (2.42, 2.70)	3.27 (3.10, 3.46)	1.91 (1.64, 2.22)	-	2.46 (2.26, 2.68)

Adjusted for maternal age at delivery, race/ethnicity, education, marital status, pre-pregnancy BMI, smoking during pregnancy, prenatal care began, insurance type, WIC, gestational diabetes mellitus, pre-pregnancy depression, prenatal depression, parity, and year of conception.

Table 3 shows the adjusted HRs and 95% CIs for CVD outcomes by both HDP subtype and onset (crude HRs and 95% CIs are shown in Supplementary Table S4). Among pregnancies with gestational hypertension, late-onset (aHR: 2.42, 95% CI: 2.32, 2.52) was more strongly associated with new chronic hypertension compared with early-onset (aHR: 1.87, 95% CI: 1.76. 1.98). Similarly, late-onset mild preeclampsia (aHR: 2.74, 95% CI: 2.06, 3.64) was more strongly associated with cardiomyopathy compared with early-onset mild preeclampsia (aHR: 0.62, 95% CI: 0.20, 1.93). Among pregnancies with severe preeclampsia, both early-onset and late-onset cases had similarly elevated risks across all outcomes. Early-onset eclampsia (aHR: 5.20, 95% CI: 3.67, 7.37) was more strongly associated with ischemic heart disease compared with late-onset eclampsia (aHR: 1.74, 95% CI: 0.91, 3.36). For superimposed preeclampsia, early-onset cases had significantly higher HRs for most CVD outcomes than late-onset cases—particularly for heart failure (early-onset aHR: 5.88, 95% CI: 5.16, 6.70 vs. late-onset aHR: 4.05, 95% CI: 3.43, 4.78), ischemic heart disease (early-onset aHR: 4.41, 95% CI: 3.96, 4.91 vs. late-onset aHR: 2.38, 95% CI: 2.04, 2.78), cerebrovascular disease or stroke (early-onset aHR: 4.01, 95% CI: 3.37, 4.78 vs. late-onset aHR: 2.65, 95% CI: 2.11, 3.33), and peripheral vascular disease (early-onset aHR: 3.89, 95% CI: 3.29, 4.62 vs. late-onset aHR: 2.10, 95% CI: 1.65, 2.69).

**Table 3:** Adjusted hazard ratios (HRs) and 95% confidence intervals (95% CIs) of CVD outcomes within 5 years after delivery by HDP subtype and onset among singleton pregnancies with a conception date between 2012 and 2017 in the linked EHR-vital statistics birth and fetal death records data in Florida.

HDP subtype and onset	HR (95% CI)						
	Heart failure	Ischemic heart disease	Cerebrovascular disease or stroke	Arrhythmia or cardiac arrest	Cardiomyopathy	Peripheral vascular disease	Hypertension
No HDP	Ref	Ref	Ref	Ref	Ref	Ref	Ref
Chronic hypertension	2.69 (2.46, 2.95)	2.28 (2.12, 2.44)	1.90 (1.70, 2.13)	1.94 (1.74, 2.17)	2.53 (2.25, 2.85)	2.30 (2.07, 2.57)	-
Gestational hypertension							
Early onset	1.61 (1.28, 2.04)	1.12 (0.91, 1.37)	1.34 (1.02, 1.75)	1.34 (1.02, 1.75)	1.33 (0.96, 1.84)	1.53 (1.17, 1.99)	1.87 (1.76, 1.98)
Late onset	2.00 (1.70, 2.35)	1.37 (1.18, 1.58)	1.21 (0.97, 1.51)	1.26 (1.02, 1.57)	1.94 (1.57, 2.40)	1.16 (0.92, 1.48)	2.42 (2.32, 2.52)
Mild preeclampsia							
Early onset	1.59 (0.92, 2.74)	1.74 (1.18, 2.57)	1.94 (1.10, 3.42)	1.55 (0.86, 2.81)	0.62 (0.20, 1.93)	1.67 (0.95, 2.95)	2.73 (2.44, 3.07)
Late onset	2.16 (1.69, 2.76)	1.46 (1.16, 1.83)	1.32 (0.92, 1.89)	1.35 (0.97, 1.88)	2.74 (2.06, 3.64)	1.27 (0.90, 1.80)	2.51 (2.36, 2.67)
Severe preeclampsia							
Early onset	3.13 (2.18, 4.49)	1.88 (1.32, 2.67)	2.79 (1.86, 4.18)	2.44 (1.57, 3.80)	4.21 (2.83, 6.27)	1.39 (0.74, 2.58)	3.44 (3.11, 3.80)
Late onset	3.44 (2.77, 4.27)	1.75 (1.39, 2.19)	1.70 (1.24, 2.34)	1.59 (1.13, 2.22)	3.78 (2.91, 4.91)	1.68 (1.18, 2.38)	3.21 (3.02, 3.42)
Eclampsia							
Early onset	7.07 (4.72, 10.59)	5.20 (3.67, 7.37)	3.26 (1.75, 6.08)	2.52 (1.25, 5.04)	4.29 (2.22, 8.28)	3.36 (1.74, 6.47)	1.65 (1.32, 2.06)
Late onset	3.10 (1.61, 5.98)	1.74 (0.91, 3.36)	2.34 (1.05, 5.23)	1.52 (0.57, 4.04)	6.43 (3.55, 11.65)	1.73 (0.65, 4.62)	2.23 (1.80, 2.76)
Superimposed preeclampsia on chronic hypertension							
Early onset	5.88 (5.16, 6.70)	4.41 (3.96, 4.91)	4.01 (3.37, 4.78)	2.83 (2.32, 3.44)	5.19 (4.36, 6.18)	3.89 (3.29, 4.62)	-
Late onset	4.05 (3.43, 4.78)	2.38 (2.04, 2.78)	2.65 (2.11, 3.33)	2.39 (1.89, 3.01)	4.37 (3.56, 5.37)	2.10 (1.65, 2.69)	-
Unspecified HDP							
Early onset	1.43 (0.74, 2.76)	2.64 (1.85, 3.76)	1.28 (0.61, 2.69)	1.96 (1.08, 3.55)	0.53 (0.13, 2.11)	1.77 (0.92, 3.42)	2.92 (2.57, 3.32)
Late onset	2.26 (1.52, 3.36)	1.55 (1.09, 2.21)	1.45 (0.84, 2.50)	1.36 (0.79, 2.35)	1.78 (1.01, 3.15)	1.88 (1.17, 3.04)	2.20 (1.97, 2.46)

Adjusted for maternal age at delivery, race/ethnicity, education, marital status, pre-pregnancy BMI, smoking during pregnancy, prenatal care began, insurance type, WIC, gestational diabetes mellitus, pre-pregnancy depression, prenatal depression, parity, and year of conception.

## Discussion

In this large retrospective cohort study, we found that HDP is strongly associated with a substantially higher risk of CVD within five years after delivery compared with women with normotensive pregnancies. Specifically, the strengths of associations varied by HDP subtype and timing of onset. Superimposed preeclampsia on chronic hypertension was associated with greatest risk of cerebrovascular disease or stroke, arrhythmia or cardiac arrest, and peripheral vascular disease, while severe preeclampsia was associated with highest elevated risk of new-onset chronic hypertension. In addition, eclampsia was associated with the greatest elevated risk of heart failure, ischemic heart disease, and cardiomyopathy. When comparing early-onset vs. late-onset for each HDP subtype, we found that early-onset eclampsia was more strongly associated with ischemic heart disease compared with late-onset eclampsia. Similarly, compared with late-onset superimposed preeclampsia, those with early-onset had a significantly higher risk of heart failure, ischemic heart disease, cerebrovascular disease or stroke, and peripheral vascular disease. On the other hand, late-onset gestational hypertension and mild preeclampsia were more strongly associated with new chronic hypertension and cardiomyopathy when compared with early-onset cases, respectively. For severe preeclampsia, early-onset and late-onset cases had similarly elevated risks across all CVD outcomes. These findings address several critical gaps in the literature by underscoring that HDP is a spectrum of conditions with distinct cardiovascular risk profiles that are associated with CVD soon after pregnancy.

Our findings align with a substantial body of literature linking HDP to elevated cardiovascular risk in general. Prior meta-analyses have shown that women with a history of preeclampsia have roughly double the risk of future cardiovascular disease (CVD) and stroke compared to normotensive pregnancies (7, 8). More recent analyses confirmed that these associations are independent of traditional risk factors: in a 2017 meta-analysis of over 6 million women, preeclampsia was linked to a 4.2-fold higher risk of future heart failure and ∼2-fold higher risks of coronary heart disease, stroke, and cardiovascular mortality (9). Our results extend this evidence by demonstrating that CVD outcomes emerge as early as five years postpartum. Consistent with Ackerman-Banks et al. (15), who observed markedly elevated rates of new-onset heart failure, stroke, and cardiomyopathy within 24 months of an HDP-affected delivery, we found significantly higher risks of these CVD outcomes within 5 years after delivery for all the HDP subtypes. Although Ackerman-Banks et al. (15) reported little difference in early postpartum ischemic heart disease and cardiac arrest/arrhythmia incidence between HDP and normotensive groups, our five-year follow-up captured a higher absolute incidence of these outcomes, revealing significantly increased risks across all HDP subtypes. Overall, the magnitude and pattern of risk in our cohort corroborate prior studies' conclusions that HDP confers an outsized risk for CVD after pregnancy (9), while also highlighting that the short-term postpartum period is a critical window for emerging CVD risk.

A unique contribution of our study is the simultaneous evaluation of HDP subtype and onset timing in relation to CVD outcomes. Previous studies rarely considered both dimensions together. Our analysis underscores the importance of HDP onset timing. Early-onset eclampsia and superimposed preeclampsia, in particular, were associated with greater short-term risk, in line with the clinical understanding that early-onset disease often signifies more severe placental and vascular pathology (27). Prior research focusing on onset timing is limited, but our results support those by Madazli et al. (27) who noted that early-onset preeclampsia is associated with worse maternal outcomes than late-onset. For severe preeclampsia, we did not observe any difference in postpartum CVD risks between early- and late-onset cases, suggesting that once preeclampsia reaches a severe threshold, the CVD risk is elevated regardless of onset, potentially due to severe placental and endothelial insult or substantial maternal cardiovascular predisposition which confers higher CVD risk whether it manifests early or late in pregnancy (30). Interestingly, late-onset gestational hypertension and mild preeclampsia were more strongly associated with new chronic hypertension and cardiomyopathy, respectively, when compared with early-onset cases. This may be due to late-onset cases for those with mild HDP more often reflecting underlying maternal cardiometabolic predispositions rather than solely placental dysfunction (31). For example, gestational hypertension emerging at term could unmask latent chronic hypertension, as these women are likely to have pre-existing hypertensive tendencies or metabolic risk factors that become apparent under the stress of late pregnancy—consequently, they have a high likelihood of persistent hypertension after delivery (28). By contrast, an “early” gestational hypertension (onset well before term) may in some cases be more tied to transient placental abnormalities that resolve postpartum, or progress to more severe HDP before term, thus not simply continuing as chronic hypertension (32). Overall, our study's findings emphasize the importance of considering both the HDP subtype and the timing of onset in postpartum CVD risk assessment as different combinations of subtype and onset convey distinct risk profiles.

These findings have important clinical and public health implications for postpartum care and CVD prevention. Women with a history of HDP represent a population that could benefit from early cardiovascular risk screening and targeted preventive interventions soon after pregnancy. In particular, those with preeclampsia and eclampsia—especially if it was early-onset or accompanied by severe features—should be counseled about their increased risk and may warrant more intensive follow-up. Early postpartum cardiovascular risk assessments (e.g., blood pressure monitoring, glucose and lipid screening at the 6–12 week visit and beyond) are increasingly recommended (14). Our results support such recommendations, as we observed clinically significant CVD events unfolding within five years of an affected pregnancy. Prompt identification of chronic hypertension, diabetes, or dyslipidemia in this high-risk group could enable timely management to mitigate progression to overt CVD. There is also a need for stratified postpartum care based on HDP subtype and timing of onset. Ultimately, recognizing HDP cases as a high-risk group provides an opportunity for early intervention—one that could improve long-term maternal cardiovascular health and is increasingly being reflected in updated clinical guidelines.

While we observed heterogeneity in CVD risk by HDP subtype and onset, the underlying biological mechanisms remain poorly understood. Future research should investigate the distinct pathophysiological pathways, such as endothelial dysfunction, inflammation, and vascular remodeling, that may differentially link early- vs. late-onset HDP subtypes to specific CVD outcomes.

This study has several strengths. It leveraged a large, diverse population-based cohort with linkage of EHR and vital statistics records, which allowed for accurate assessment of pregnancies, HDP subtype and onset, and CVD outcomes within five years postpartum. The granularity of our HDP classification is a particular strength, enabling a detailed analysis of how specific HDP subtypes and onset are associated with CVD within five years after delivery. These design features enhance confidence in the validity and general relevance of our findings.

Several limitations should be acknowledged. First, as an observational study, our analysis is subject to residual confounding. We adjusted for many pre-pregnancy and peripartum covariates, yet unmeasured factors (e.g., genetic predisposition) may have influenced both HDP occurrence and CVD outcomes. Second, the associations we observed should not be interpreted as evidence that HDP causes CVD, but it is more likely that the physiological stress of pregnancy simply reveals underlying risk rather than directly causing disease. Third, despite using linked electronic health records and vital statistics data with multidomain clinical information, outcome and exposure misclassification remains a possibility. Retrospective classification of HDP subtypes is inherently challenging, particularly given diagnostic overlap and evolving clinical presentations. We attempted to mitigate this by incorporating blood pressure values, medication records, and laboratory results where available; however, inaccuracies in diagnostic coding and documentation may persist. Fourth, our cohort was region-specific, so the generalizability to other populations or settings—especially those with different racial/ethnic compositions or access to care—may be limited.

## Conclusions

This study highlights important heterogeneity in the cardiovascular sequelae of HDP by both subtype and timing of onset. Our findings highlight the importance of considering a pregnancy complicated by HDP as an early warning sign for a woman's cardiovascular health. The elevated risks among early-onset cases, especially for severe HDP subtypes, underscore the importance of early identification and postpartum surveillance in this high-risk population.

## Data Availability

The data analyzed in this study is subject to the following licenses/restrictions: Florida vital statistic birth records and fetal death records data can be requested from Bureau of Vital Statistics, Florida Department of Health (http://www.floridahealth.gov/certificates/). EHR data can be requested from the OneFlorida+ Clinical Research Network. Requests to access these datasets should be directed to http://www.floridahealth.gov/certificates/ and https://onefl.net/.
