# Supplementary material for: Subtypes and onset of hypertensive disorders of pregnancy and cardiovascular disease within 5 years after delivery
Source: Front Cardiovasc Med. 2026 Feb 19;13:1701507. doi: 10.3389/fcvm.2026.1701507 (PMC12960569; doi:10.3389/fcvm.2026.1701507)
Supplement: Supplementary file 1 [file Datasheet1.docx]

| **Supplemental Table 1.** ICD-9-CM and ICD-10-CM codes for HDP, CVD, GDM, and depression diagnoses. | | |
| --- | --- | --- |
|  | ICD-9-CM | ICD-10-CM |
| HDP |  |  |
| Chronic hypertension | 401.x, 402.x, 403.x, 404.x, 405.x, 642.0x, 642.1x, 642.2x | I10.x, I11.x, I12.x, I13.x, I15.x, I16.x, O10.x |
| Gestational hypertension | 642.3x | O13.x |
| Preeclampsia with mild features | 642.4x | O14.0x |
| Preeclampsia with severe features | 642.5x | O14.1x, O14.2x |
| Eclampsia | 642.6x | O15.x |
| Superimposed preeclampsia on chronic hypertension | 642.7x | O11.x |
| Unspecified HDP | 642.9x | O16.x |
| CVD |  |  |
| Heart failure | 428.0,428.1,428.20,428.21,428.22,428.23,428.30,428.31,428.32,428.33,428.40,428.41,428.42,428.43,428.9,402.01,402.11,402.91,404.01,404.03,404.11,404.13,404.91,404.93,402.01,402.11,402.91 | I09.81, I11.0, I13.0, I13.2, I50.1,I50.20, I50.21, I50.22, I50.23, I50.30, I50.31, I50.32, I50.33,I50.40, I50.41, I50.42, I50.43, I50.9,I50.810, I50.811, I50.812, I50.813,I50.814, I50.82, I50.83, I50.84, I50.8 |
| Ischemic heart disease | 410.x,411.1,411.8,4111,4118 | I20.0, I20.1, I20.8, I20.9, I21.01, I21.02, I21.09, I21.11, I21.19,I21.21, I21.29, I21.3, I21.4, I21.9, I21.A1, I21.A9, I22.0, I22.1, I22.2, I22.8, I22.9, I23.0, I23.1,I23.2, I23.3, I23.4, I23.5, I23.6, I23.7, I23.8, I24.0, I24.1, I24.8, I24.9, I25.10, I25.110, I25.111, I25.118, I25.119, I25.2, I25.3, I25.41, I25.42, I25.5, I25.6, I25.700, I25.701, I25.708, I25.709, I25.710, I25.711, I25.718, I25.719, I25.720, I25.721, I25.728, I25.729, I25.730, I25.731,I25.738, I25.739, I25.750, I25.751, I25.758, I25.759, I25.760, I25.761, I25.768, I25.769, I25.790, I25.791, I25.798, I25.799, I25.810, I25.811, I25.812, I25.82, I25.83, I25.84, I25.89, I25.9 |
| Cerebrovascular disease or stroke | 430, 431, 432.x, 433.x, 434.x,435.x, 436, 437.x, 671.5, 674.0,997.02 | G45.0, G45.1, G45.2, G45.3, G45.8, G45.9,G46.0, G46.1, G46.2, G46.3, G46.4, G46.5, G46.6, G46.7, G46.8,G97.31, G97.32, I60.00, I60.01,I60.02, I60.10, I60.11, I60.12,I60.20, I60.21, I60.22, I60.30,I60.31, I60.32, I60.4, I60.50, I60.51,I60.52, I60.6, I60.7, I60.8, I60.9,I61.0, I61.1, I61.2, I61.3, I61.4,I61.5, I61.6, I61.8, I61.9, I63.00,I63.011, I63.012, I63.013, I63.019,I63.02, I63.031, I63.032, I63.039,I63.09, I63.10, I63.111, I63.112,I63.119, I63.12, I63.131, I63.132,I63.133, I63.139, I63.19, I63.20,I63.211, I63.212, I63.213, I63.219,I63.22, I63.231, I63.232, I63.233,I63.239, I63.29, I63.30, I63.311,I63.312, I63.313, I63.319, I63.321,I63.322, I63.323, I63.329, I63.331,I63.332, I63.333, I63.339, I63.341,I63.342, I63.343, I63.349, I63.39,I63.40, I63.411, I63.412, I63.413,I63.419, I63.421, I63.422, I63.423,I63.429, I63.431, I63.432, I63.433,I63.439, I63.441, I63.442, I63.443,I63.449, I63.49, I63.50, I63.511,I63.512, I63.513, I63.519, I63.521,I63.522, I63.523, I63.529, I63.531,I63.532, I63.533, I63.539, I63.541,I63.542, I63.543, I63.549, I63.59,I63.6, I63.8, I63.9, I66.01, I66.02,I66.03, I66.09, I66.11, I66.12,I66.13, I66.19, I66.21, I66.22,I66.23, I66.29, I66.3, I66.8, I66.9,I67.841, I67.848, I97.810, I97.811,I97.820, I97.821, I60.2, I63.033,I63.113, I62.9, I65.1, I65.29, I65.09,I6781, I67.2, I67.82, I67.89, I67.4,I67.1, I67.7, I67.5, I67.6, I67.9,I67.848, O87.3, O99.4 |
| Arrhythmia or cardiac arrest | 427.31,427.32, 427.41, 427.42,427.5 | I49.01, I40.92, I46.9, I48.0, I48.1, I48.2, I48.91, I48.4, I48.92 |
| Cardiomyopathy | 674.5x,425.x | O90.3, I42.1, I42.2, I42.4, I42.5, I42.6, I42.7, I42.8, I42.9 |
| Peripheral vascular disease | 440.0, 440.1, 440.2, 440.20, 440.21, 440.22, 440.23, 440.29, 440.4, 443.8, 443.81, 443.82, 443.89, 443.9 | E08.51, E08.52, E09.51, E09.52, E10.51, E10.52, E11.51, E11.52, E13.51, E13.52, I70.0, I70.1, I70.201, I70.202, I70.203, I70.208, I70.209, I70.211, I70.212, I70.213, I70.218, I70.219, I70.221, I70.222, I70.223, I70.228, I70.229, I70.231, I70.232, I70.233, I70.234, I70.235, I70.238, I70.239, I70.241, I70.242, I70.243, I70.244, I70.245, I70.248, I70.249, I70.25, I70.291, I70.292, I70.293, I70.298, I70.299, I70.92, I73.81, I73.89, I73.9, I79.1, I79.8 |
| Hypertension | 401.x, 402.x, 403.x, 404.x, 405.x | I10.x, I11.x, I12.x, I13.x, I15.x, I16.x |
| GDM | 648.8x | O24.4x |
| Depression | 296.2x, 296.3x, 311 | F32.x, F33.x, F34.1x |

| **Supplemental Table 2.** LOINC codes and cut points for laboratory tests to determine proteinuria. | | |
| --- | --- | --- |
| Laboratory Test | LOINC Codes | Cut Point for Proteinuria |
| Urine Protein (Random or Dipstick Test) | 2887-8, 2888-6, 32209-9, 32551-4, 50561-0, 21482-5, 20454-5, 5804-0 | ≥ 2 or ≥ 100mg/dL |
| Urine Protein/Creatinine Ratio | 9318-7, 32294-1 | ≥ 300 mg/g |
| 24-Hour Urine Protein | 2889-4, 13801-6 | ≥ 300 mg per 24h |
| Urine Albumin/Creatinine Ratio | 14959-1 | ≥ 70 mg/g |
| 24-Hour Urine Albumin | 30003-8 | ≥ 300 mg per 24h |

| **Supplemental Table 3.** A rule-based phenotyping algorithm to determine HDP subtypes. | | | | | | | |
| --- | --- | --- | --- | --- | --- | --- | --- |
| HDP Subtype | Phenotyping Algorithm | | | | | | |
|  |  | | | | | | |
| Chronic Hypertension | Presence of chronic hypertension diagnoses (EHR) | OR | Presence of unspecified HDP diagnoses or non-presence of any HDP diagnoses (EHR) | | | | |
|  |  |  | AND | | | | |
|  |  |  | Blood pressure ≥140/90mmHg (on ≥ 2 occasions taken ≥ 4 hours apart) or ≥ 160/110mmHg (≥ 1 occasion) between 1 year before conception to 20^th^ week of gestation (EHR) | OR | Prescription of antihypertensive medications between 1 year before conception to 20^th^ week of gestation (EHR) | OR | Presence of chronic hypertension record and non-presence of gestational hypertension, preeclampsia, or eclampsia records (VSBR/VSFDR) |
|  | | | | | | | |
| Gestational Hypertension | Presence of gestational hypertension diagnoses (EHR) | OR | Presence of unspecified HDP diagnoses or non-presence of any HDP diagnoses (EHR) | | | | |
|  |  |  | AND | | | | |
|  |  |  | Non-presence of proteinuria between 20^th^ week of gestation to delivery (EHR) | | | | |
|  |  |  | AND | | | | |
|  |  |  | Blood pressure ≥140/90mmHg (on ≥ 2 occasions taken ≥ 4 hours apart) or ≥ 160/110mmHg (≥ 1 occasion) between 20^th^ week of gestation to delivery (EHR) | OR | Prescription of antihypertensive medications between 20^th^ week of gestation to delivery (EHR) | | |
|  | | | | | | | |
| Mild Preeclampsia | Presence of preeclampsia with mild features diagnoses (EHR) | OR | Presence of unspecified HDP diagnoses or gestational hypertension diagnoses (EHR) | OR | Non-presence of any HDP diagnoses (EHR) | | |
|  |  |  |  |  | AND | | |
|  |  |  |  |  | Presence of proteinuria between 20^th^ week of gestation to delivery (EHR) | | |
|  |  |  | AND |  | AND | | |
|  |  |  | Presence of proteinuria between 20^th^ week of gestation to delivery (EHR) |  | Blood pressure ≥140/90mmHg (on ≥ 2 occasions taken ≥ 4 hours apart) or ≥ 160/110mmHg (≥ 1 occasion) between 20^th^ week of gestation to delivery (EHR) | OR | Prescription of antihypertensive medications between 20^th^ week of gestation to delivery (EHR) |
|  | | | | | | | |
| Severe Preeclampsia | Presence of preeclampsia with severe features diagnoses (EHR) | OR | Presence of preeclampsia with mild features diagnoses (EHR) | OR | Non-presence of any HDP diagnoses (EHR) | | |
|  |  |  |  |  | AND | | |
|  |  |  | AND |  | Presence of proteinuria between 20^th^ week of gestation to delivery (EHR) | | |
|  |  |  | Blood pressure ≥ 160/110mmHg (≥ 1 occasion) between 20^th^ week of gestation to delivery (EHR) |  | AND | | |
|  |  |  |  |  | Blood pressure ≥ 160/110mmHg (≥ 1 occasion) between 20^th^ week of gestation to delivery (EHR) | | |
|  |  |  |  |  |  |  |  |
| Eclampsia | Presence of eclampsia diagnoses (EHR) | OR | Presence of unspecified HDP diagnoses or gestational hypertension diagnoses (EHR) | | | | |
|  |  |  | AND | | | | |
|  |  |  | Presence of eclampsia records (VSBR/VSFDR) | | | | |
|  | | | | | | | |
| Superimposed Preeclampsia on Chronic Hypertension | Presence of superimposed preeclampsia on chronic hypertension diagnoses (EHR) | OR | Presence of preeclampsia with mild features or preeclampsia with severe features diagnoses (EHR) | | | | |
|  |  |  | AND | | | | |
|  |  |  | Blood pressure ≥140/90mmHg (on ≥ 2 occasions taken ≥ 4 hours apart) or ≥ 160/110mmHg (≥ 1 occasion) between 1 year before conception to 20^th^ week of gestation (EHR) | OR | Prescription of antihypertensive medications between 1 year before conception to 20^th^ week of gestation (EHR) | OR | Presence of chronic hypertension records (VSBR/VSFDR) |
|  |  |  |  |  |  |  |  |

| **Supplemental Table 4.** Crude hazard ratios (HRs) and 95% confidence intervals (95% CIs) of CVD outcomes within 5 years after delivery by HDP subtype and onset among singleton pregnancies with a conception date between 2012-2017 in the linked EHR-vital statistics birth and fetal death records data in Florida. | | | | | | | |
| --- | --- | --- | --- | --- | --- | --- | --- |
|  | Heart failure | Ischemic heart disease | Cerebrovascular disease or stroke | Arrhythmia or cardiac arrest | Cardiomyopathy | Peripheral vascular disease | Hypertension |
| No HDP | Ref | Ref | Ref | Ref | Ref | Ref | Ref |
| Chronic hypertension | 3.95 (3.62, 4.31) | 3.50 (3.27, 3.74) | 2.50 (2.24, 2.78) | 2.59 (2.33, 2.88) | 3.72 (3.32, 4.17) | 3.22 (2.91, 3.56) | - |
| Gestational hypertension |  |  |  |  |  |  |  |
| Early onset | 1.80 (1.42, 2.27) | 1.22 (0.99, 1.50) | 1.44 (1.10, 1.89) | 1.40 (1.06, 1.83) | 1.50 (1.09, 2.07) | 1.60 (1.23, 2.09) | 2.04 (1.93, 2.17) |
| Late onset | 2.22 (1.89, 2.61) | 1.50 (1.30, 1.74) | 1.30 (1.04, 1.62) | 1.32 (1.06, 1.63) | 2.13 (1.72, 2.62) | 1.20 (0.95, 1.52) | 2.65 (2.55, 2.77) |
| Mild preeclampsia |  |  |  |  |  |  |  |
| Early onset | 1.99 (1.15, 3.43) | 2.14 (1.46, 3.15) | 2.03 (1.15, 3.57) | 1.80 (0.99, 3.25) | 0.74 (0.24, 2.31) | 2.16 (1.23, 3.82) | 3.57 (3.19, 4.00) |
| Late onset | 2.40 (1.88, 3.06) | 1.49 (1.18, 1.87) | 1.24 (0.87, 1.76) | 1.39 (1.00, 1.93) | 2.90 (2.19, 3.85) | 1.41 (1.00, 1.99) | 2.79 (2.62, 2.97) |
| Severe preeclampsia |  |  |  |  |  |  |  |
| Early onset | 3.72 (2.59, 5.33) | 2.06 (1.45, 2.93) | 3.28 (2.19, 4.91) | 2.64 (1.70, 4.10) | 5.03 (3.39, 7.48) | 1.46 (0.78, 2.72) | 3.93 (3.56, 4.34) |
| Late onset | 3.76 (3.03, 4.66) | 1.78 (1.42, 2.23) | 1.84 (1.34, 2.53) | 1.60 (1.15, 2.24) | 4.15 (3.20, 5.39) | 1.62 (1.14, 2.30) | 3.39 (3.18, 3.60) |
| Eclampsia |  |  |  |  |  |  |  |
| Early onset | 10.08 (6.74, 15.08) | 7.08 (5.00, 10.03) | 4.47 (2.40, 8.32) | 3.46 (1.73, 6.93) | 6.09 (3.16, 11.74) | 4.32 (2.24, 8.31) | 2.43 (1.95, 3.03) |
| Late onset | 3.50 (1.82, 6.74) | 1.88 (0.98, 3.61) | 2.56 (1.15, 5.72) | 1.66 (0.62, 4.42) | 6.95 (3.84, 12.58) | 1.84 (0.69, 4.90) | 2.49 (2.01, 3.08) |
| Superimposed preeclampsia on chronic hypertension |  |  |  |  |  |  |  |
| Early onset | 9.83 (8.69, 11.13) | 7.37 (6.65, 8.17) | 5.37 (4.53, 6.35) | 3.91 (3.23, 4.73) | 8.68 (7.34, 10.25) | 6.19 (5.26, 7.29) | - |
| Late onset | 5.79 (4.92, 6.81) | 3.36 (2.88, 3.91) | 3.14 (2.51, 3.94) | 2.96 (2.36, 3.72) | 6.19 (5.06, 7.57) | 2.90 (2.27, 3.70) | - |
| Unspecified HDP |  |  |  |  |  |  |  |
| Early onset | 1.88 (0.98, 3.63) | 3.48 (2.44, 4.96) | 1.61 (0.76, 3.38) | 2.44 (1.35, 4.42) | 0.68 (0.17, 2.73) | 2.21 (1.15, 4.26) | 3.90 (3.43, 4.44) |
| Late onset | 2.61 (1.76, 3.88) | 1.78 (1.25, 2.53) | 1.52 (0.88, 2.62) | 1.47 (0.85, 2.54) | 2.03 (1.15, 3.58) | 2.13 (1.32, 3.43) | 2.57 (2.30, 2.87) |


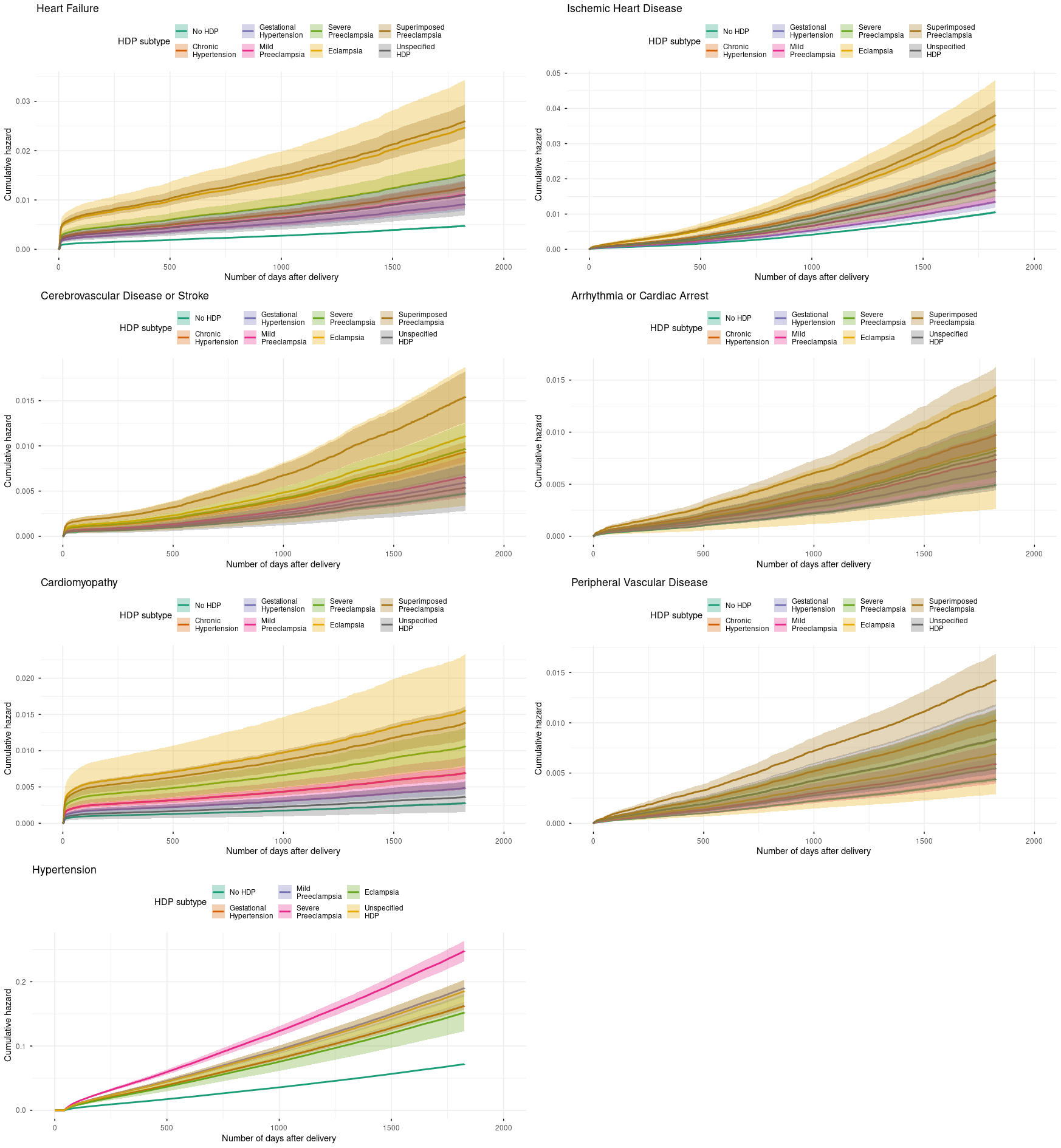


**Supplemental Figure 1.** Cumulative hazard curves of CVD outcomes within 5 years after delivery by HDP subtype among singleton pregnancies with a conception date between 2012-2017 in the linked EHR-vital statistics birth and fetal death records data in Florida.
